# How to Keep Your Friends

**Published:** 1881-11

**Authors:** 


### HOW TO KEEP YOUR FRIENDS.

In the first place, don't be too exacting. If your friend doesn't come to see you as often as you wish, or if she is dilatory about answering your letters, don't make up your mind at once that she has grown cold or indifferent, and above all don't everwhelm her with reproaches. Rest assured that there is no more certain way of killing a friendship than by exactions and upbraidings. It is quite possible that your friend may have other duties and engagements whose performance employs the very time you would claim, and instead of being neglected, you are only waiting your turn. Perhaps she comes to you in her rare intervals of leisure to be rested and cheered and helped by your affection and sympathy. But is she likely to find cheer or comfort in your society if you meet her with doubts, with coldness, or with a sense of injury, and insist on a full account of how she has spent her time, and whether she could not possibly have come before. In nine cases out of ten she will go away feeling that she is injured by what you consider affection, and that your friendship is a trouble rather than a help.

